# Supplementary material for: Comparison of Contributors to Mortality Differences in SLE Patients with Different Initial Disease Activity: A Larger Multicenter Cohort Study
Source: J Clin Med. 2023 Jan 30;12(3):1061. doi: 10.3390/jcm12031061 (PMC9918091; doi:10.3390/jcm12031061)
Supplement: Supplementary file 1 [file jcm-12-01061-s001.zip › jcm-2187965-SI.pdf]

# SUPPLEMENTARY MATERIALS

**Table S1.** The number and percentage of main manifestations in specific organ involvements.

| Organ involvements | N | % |
|---|---|---|
| **Mucocutaneous** | 1589 | |
| Skin eruption | 1385 | 87.2 |
| Mucosal ulceration | 289 | 18.2 |
| Alopecia | 256 | 16.1 |
| Cutaneous vasculitis | 237 | 14.9 |
| Others | 13 | 0.8 |
| **Neuropsychiatric** | 151 | |
| Headache | 51 | 33.8 |
| Epilepsy | 49 | 32.5 |
| Acute confusional state | 25 | 16.6 |
| Mood disorder (depression/ mania) | 21 | 13.9 |
| Cerebral vasculitis | 12 | 7.9 |
| Psychosis | 10 | 6.6 |
| Others | 11 | 7.3 |
| **Musculoskeletal** | 1335 | |
| Arthritis | 1305 | 97.8 |
| Myositis | 63 | 4.7 |
| **Cardiopulmonary** | 523 | |
| Serositis | 413 | 79.0 |
| Interstitial lung disease | 110 | 21.0 |
| Myocarditis | 22 | 4.2 |
| Pulmonary arterial hypertension | 16 | 3.1 |
| Others | 17 | 3.3 |
| **Gastrointestinal** | 836 | |
| Elevated liver enzymes | 794 | 95.0 |
| Hepatitis | 56 | 6.7 |
| Peritonitis | 19 | 2.3 |
| Malabsorption | 17 | 2.0 |
| Mesenteric vasculitis | 7 | 0.8 |
| Ascites | 7 | 0.8 |
| Otherts | 16 | 1.9 |
| **Ocular** | 11 | |
| Retinitis | 4 | 36.4 |
| Keratitis | 3 | 27.3 |
| Optic neuritis | 2 | 18.2 |
| Xerophthalmia | 2 | 18.2 |
| **Renal** | 1591 | |
| Proteinuria | 1140 | 71.7 |
| Decrease of creatinine clearance rate | 830 | 52.2 |
| Hypertension (renal related) | 286 | 18.0 |
| Biopsy-proved lupus nephritis | 135 | 8.5 |
| Active urinary sediment | 67 | 4.2 |
| Hematuresis | 35 | 2.2 |
| Others | 4 | 0.3 |
| **Hematological** | 1150 | |
| Thrombocytopenia | 733 | 63.7 |
| Leukopenia | 631 | 54.9 |
| Hemolytic anemia | 103 | 9.0 |

**Table S2.** The association between items in SLEDAI score on admission and death in SLE patients.

| Variables | Survival No. (%) | Death No. (%) | HR (95% CI) | Adjusted HR (95% CI) [a] |
|---|---|---|---|---|
| Seizure | | | | |
| No | 2149(98.3) | 246(95.0) | 1.00 | 1.00 |
| Yes | 38(1.7) | 13(5.0) | 2.73(1.57, 4.78) | 2.97(1.69, 5.19) |
| Psychosis | | | | |
| No | 1443(66.0) | 164(63.3) | 1.00 | 1.00 |
| Yes | 744(34.0) | 95(36.7) | 1.23(0.95, 1.58) | 1.27(0.98, 1.65) |
| Organic brain syndrome | | | | |
| No | 1965(89.8) | 237(91.5) | 1.00 | 1.00 |
| Yes | 222(10.2) | 22(8.5) | 0.93(0.60, 1.44) | 0.92(0.60, 1.43) |
| Visual disturbance | | | | |
| No | 2163(98.9) | 245(94.6) | 1.00 | 1.00 |
| Yes | 24(1.1) | 14(5.4) | 3.67(2.13, 6.34) | 3.69(2.14, 6.37) |
| Cranial nerve disorder | | | | |
| No | 2143(98.0) | 249(96.1) | 1.00 | 1.00 |
| Yes | 44(2.0) | 10(3.9) | 2.09(1.11, 3.93) | 1.98(1.05, 3.73) |
| Lupus headache | | | | |
| No | 2019(92.3) | 218(84.2) | 1.00 | 1.00 |
| Yes | 168(7.7) | 41(15.8) | 2.19(1.57, 3.06) | 2.15(1.54, 3.01) |
| Cerebrovascular accident | | | | |
| No | 2167(99.1) | 248(95.8) | 1.00 | 1.00 |
| Yes | 20(0.9) | 11(4.2) | 3.78(2.07, 6.92) | 3.86(2.10, 7.09) |
| Vasculitis | | | | |
| No | 2091(95.6) | 242(93.4) | 1.00 | 1.00 |
| Yes | 96(4.4) | 17(6.6) | 1.53(0.94, 2.50) | 1.56(0.95, 2.56) |
| Arthritis | | | | |
| No | 1584(72.4) | 180(69.5) | 1.00 | 1.00 |
| Yes | 603(27.6) | 79(30.5) | 1.18(0.90, 1.53) | 1.21(0.92, 1.58) |
| Myositis | | | | |
| No | 2158(98.7) | 252(97.3) | 1.00 | 1.00 |
| Yes | 29(1.3) | 7(2.7) | 2.20(1.04, 4.67) | 2.19(1.03, 4.65) |
| Urinary casts | | | | |
| No | 1877(85.8) | 201(77.6) | 1.00 | 1.00 |
| Yes | 310(14.2) | 58(22.4) | 1.80(1.34, 2.41) | 1.96(1.46, 2.63) |
| Hematuria | | | | |
| No | 1196(54.7) | 133(51.4) | 1.00 | 1.00 |
| Yes | 991(45.3) | 126(48.6) | 1.16(0.91, 1.48) | 1.23(0.96, 1.57) |
| Proteinuria | | | | |
| No | 1711(78.2) | 211(81.5) | 1.00 | 1.00 |
| Yes | 476(21.8) | 48(18.5) | 0.74(0.54, 1.01) | 0.76(0.55, 1.04) |
| Pyuria | | | | |
| No | 2012(92.0) | 241(93.1) | 1.00 | 1.00 |
| Yes | 175(8.0) | 18(6.9) | 0.81(0.50, 1.31) | 0.80(0.50, 1.30) |
| Rash | | | | |
| No | 1397(63.9) | 145(56.0) | 1.00 | 1.00 |
| Yes | 790(36.1) | 114(44.0) | 1.33(1.04, 1.70) | 1.46(1.14, 1.88) |
| Alopecia | | | | |
| No | 1140(52.1) | 130(50.2) | 1.00 | 1.00 |
| Yes | 1047(47.9) | 129(49.8) | 1.21(0.94, 1.54) | 1.23(0.96, 1.57) |
| Mucosal ulcers | | | | |
| No | 2058(94.1) | 243(93.8) | 1.00 | 1.00 |
| Yes | 129(5.9) | 16(6.2) | 1.24(0.75, 2.06) | 1.21(0.73, 2.01) |
| Pleurisy | | | | |
| No | 1943(88.8) | 219(84.6) | 1.00 | 1.00 |
| Yes | 244(11.2) | 40(15.4) | 1.47(1.05, 2.06) | 1.37(0.97, 1.92) |
| Pericarditis | | | | |
| No | 1681(76.9) | 170(65.6) | 1.00 | 1.00 |
| Yes | 506(23.1) | 89(34.4) | 1.81(1.40, 2.35) | 1.85(1.43, 2.40) |
| Low complement | | | | |

| | | | | |
|---|---|---|---|---|
| No | 1522(69.6) | 198(76.4) | 1.00 | 1.00 |
| Yes | 665(30.4) | 61(23.6) | 0.69(0.51, 0.91) | 0.69(0.52, 0.93) |
| Increased DNA binding | | | | |
| No | 1486(67.9) | 195(75.3) | 1.00 | 1.00 |
| Yes | 701(32.1) | 64(24.7) | 0.77(0.58, 1.03) | 0.79(0.60, 1.05) |
| Fever | | | | |
| No | 1547(70.7) | 150(57.9) | 1.00 | 1.00 |
| Yes | 640(29.3) | 109(42.1) | 1.76(1.37, 2.25) | 1.75(1.37, 2.25) |
| Thrombocytopenia | | | | |
| No | 1876(85.8) | 221(85.3) | 1.00 | 1.00 |
| Yes | 311(14.2) | 38(14.7) | 1.00(0.71, 1.41) | 0.96(0.68, 1.35) |
| Leukopenia | | | | |
| No | 1185(54.2) | 148(57.1) | 1.00 | 1.00 |
| Yes | 1002(45.8) | 111(42.9) | 0.96(0.75, 1.23) | 1.04(0.81, 1.33) |

[a] Adjusted for sex (male = 1, female = 0), age (continuous), comorbidities (yes = 1, no = 0), glucocorticoids treatment (yes = 1, no = 0), immunosuppressive treatment (yes = 1, no = 0). SLEDAI: SLE disease activity index 2000.
